# A comparative study of intraoperative frozen section and alpha defensin lateral flow test in the diagnosis of periprosthetic joint infection

**DOI:** 10.1080/17453674.2019.1567153

**Published:** 2019-01-23

**Authors:** Irene K Sigmund, Johannes Holinka, Susanna Lang, Sandra Stenicka, Kevin Staats, Gerhard Hobusch, Bernd Kubista, Reinhard Windhager

**Affiliations:** a Medical University of Vienna, Department of Orthopaedics and Trauma Surgery, Vienna;; b Medical University of Vienna, Department of Pathology, Vienna, Austria

## Abstract

Background and purpose — For decision-making (aseptic vs. septic), surgeons rely on intraoperatively available tests when a periprosthetic joint infection (PJI) cannot be confirmed or excluded preoperatively. We compared and evaluated the intraoperative performances of the frozen section and the alpha defensin lateral flow test in the diagnosis of PJI.

Patients and methods — In this prospective study, consecutive patients with indicated revision surgery after arthroplasty were included. Patients were classified as having PJI using the MusculoSkeletal Infection criteria. The presence of alpha defensin was determined using the lateral flow test intraoperatively. During revision surgery, tissue samples were harvested for frozen and permanent section. Analysis of diagnostic accuracy was based on receiver-operating characteristics.

Results — 101 patients (53 hips, 48 knees) were eligible for inclusion. Postoperatively, 29/101 patients were diagnosed with PJI, of which 8/29 cases were definitely classified as septic preoperatively. Of the remainder 21 septic cases, the intraoperative alpha defensin test and frozen section were positive in 13 and 17 patients, respectively. Sensitivities of the alpha defensin test and frozen section were 69% and 86%, respectively. The area under the curves of both tests showed a statistically significant difference (p = 0.006).

Interpretation — The frozen section showed a significantly higher performance compared with the alpha defensin test and a near perfect concordance with the definitive histology, and therefore remains an appropriate intraoperative screening test in diagnosing PJI. Although the sensitivity of the alpha defensin test was lower compared with that of frozen section, this test is highly specific for confirming the diagnosis of PJI.

Despite various available diagnostic test methods, diagnosing periprosthetic joint infection (PJI) remains very challenging. When results are not available or cannot be interpreted preoperatively, an intraoperative screening test is required to confirm or exclude PJI.

Some studies have shown good concordance between the intraoperative evaluation of frozen tissue samples and paraffin-embedded permanent sections ranging from 95% to 100% (Della Valle et al. 1999, Musso et al. 2003, Bori et al. 2007, Stroh et al. 2012, Kwiecien et al. 2017). In the recent study by Kwiecien et al. (2017), the sensitivity and specificity of intraoperative frozen section was 74% and 99%, respectively. This is in line with the reported 73% and 95% of permanent sections by Morawietz et al. (2009). Therefore, frozen sections show comparable results to the definitive histopathology and could be useful for confirming the presence or absence of PJI intraoperatively.

On the other hand, in recent years attention was also focused on the biomarker alpha defensin, which is released by neutrophils into the synovial fluid and induces rapid microorganism death due to depolarization of the cell membrane (Ganz et al. 1985, Chalifour et al. 2004). The alpha defensin lateral flow test showed sensitivities ranging from 69% to 92% and high specificities close to 100% (Sigmund et al. 2017, Gehrke et al. 2018, Renz et al. 2018). While results of the quantitative alpha defensin test (ELISA) are available within one day, the lateral flow test is characterized by ease of use and quick results within 10 minutes, thus rendering its intraoperative use frequent.

We evaluated the intraoperative performance of the alpha defensin lateral flow test and the histopathological analysis of frozen tissue samples using the Musculoskeletal Infection Society (MSIS) criteria (Parvizi and Gehrke 2014). In addition, the results of both diagnostic methods were compared.

## Patients and methods

### Study design

This prospective cohort study was conducted in a tertiary healthcare center providing advanced specialty care to patients with PJI with multidisciplinary collaborations.

This study includes some data (31 patients) from a previously published study analyzing the sensitivity and specificity of qualitative alpha defensin in all kinds of revision surgery (including the second stage of a 2-stage revision and spacer exchanges, which were excluded in this study) (Sigmund et al. 2017).

### Study population

Patients with an indicated revision surgery between January 2016 and February 2018 were eligible for inclusion. Inclusion criteria comprised an indicated revision surgery after TJA, sufficient synovial fluid derived from the affected joint, a conclusive alpha defensin lateral flow test, a frozen section, and permanent histopathology. Samples with obvious contaminated joint fluid and/or failed periprosthetic tissue samples for pathohistological analysis were excluded. Further exclusion criteria (and the difference from the previously published study) were surgery within the last 6 weeks, a joint aspiration with a cement spacer in place, the second stage of 2-stage revision, or a resection arthroplasty.

## Definition of infection

In accordance with the MSIS criteria (Parvizi and Gehrke 2014), PJI was diagnosed when a sinus tract communicating with the joint was present, 2 or more periprosthetic cultures grew phenotypically identical organisms, or at least 3 of the following 5 minor criteria were present: (i) elevated serum C-reactive protein (CRP) (acute: > 100 mg/L or chronic: > 10 mg/L), (ii) elevated synovial fluid white blood cell (acute: WBC > 10,000/µL or chronic: WBC > 3,000/µL), (iii) elevated synovial fluid polymorphonuclear neutrophil percentage (acute: PMN% > 90% or chronic: PMN% > 80%), (iv) positive histological analysis of periprosthetic tissue, and/or (v) a single positive culture. The serum erythrocyte sedimentation rate (ESR), which has low sensitivity and specificity for PJI, was not routinely determined in our institution (Piper et al. 2010). We did not evaluate leucocyte esterase colorimetric test strips in our study as Deirmengian et al. (2015) have shown that alpha defensin is a better test.

### Determination of diagnostic tests

For all patients, a standardized diagnostic workup was performed. First, blood samples were collected to assess the serum CRP levels. In line with proceedings of the International Consensus Meeting (Parvizi et al. 2013), a cut-off of 100 mg/L (acute) or 10 mg/L (chronic) was chosen as positive with suspicion of infection (systemic or local).

Preoperatively, synovial fluid was aspirated under sterile conditions. 1 mL was placed into a vial containing ethylenediaminetetraacetic acid (EDTA) for quantification of the erythrocyte and leukocyte count as well as granulocyte percentage. Another 1 mL was sent for microbiological investigations and processed per standard laboratory protocol with cultures held for 14 days (Butler-Wu et al. 2011, Parvizi et al. 2013).

For alpha defensin measurements, synovial joint fluid was aspirated in the operation room before arthrotomy by direct needle aspiration (Diagnostics 2013). For qualitative alpha defensin testing, the Synovasure™ test (Zimmer Inc., Warsaw, IN, USA) was used according to the manufacturer’s instructions. The synovial fluid was processed and the qualitative result (i.e., infection present yes or no) was read after 10 min. The control line (“C”) had to appear; otherwise the test was considered inconclusive.

Intraoperatively, at least 3 periprosthetic tissue samples were sent for microbiological investigations and processed per standard laboratory protocol with cultures held for 14 days (Schafer et al. 2008, Butler-Wu et al. 2011, Parvizi et al. 2013). All the explanted prosthetic components were also sent for sonication culture analysis.

In all cases, at least 1 tissue sample (median: 3, range 1–8) for frozen section was taken from 1 of several sites at the time of revision surgery. For frozen section, multiple sections or 1 representative area from each sample were prepared at ™20°C in the Microm HM 550 cryocut (Thermo Fisher Scientific, Walldorf, Germany) for about 3 min. Sections of 3 µm thickness were cut and stained with hematoxylin and eosin. The samples were analyzed and evaluated under high power (×400 magnification). The diameter of the visual field was 0.625 mm; hence the visual field was 0.307 mm^2^. At least 40 high-powered fields (HPFs) were evaluated for each slice. The samples were interpreted by 1 of 3 senior pathologists specialized in musculoskeletal infections. After about 15–25 min, the pathologist communicated the results to the orthopedic surgeon using the intercom system.

For definitive histopathological analysis, samples for frozen section and additional periprosthetic tissue specimens (median 5, range 2–12) were obtained intraoperatively in all patients. The samples were sent to histopathological analysis and fixed in 4.5% formaldehyde for 12 hours, paraffin embedded and also stained with hematoxylin and eosin. The samples were analyzed and classified according to the Krenn criteria by default (Morawietz et al. 2009) by 1 of the 3 pathologists. If the number of neutrophil granulocytes was > 23 in 10 HPFs the sample was classified as positive.

### Statistics

Analysis of diagnostic accuracy is based on the receiver-operating characteristic (ROC). Sensitivity, specificity, area under the ROC curve (AUC), positive and negative likelihood ratios (LR + and LR–, respectively), accuracy (calculated as the number of correct classifications/number of total classifications), positive (PPV) and negative predictive value (NPV), and their 95% confidence intervals (CI) were calculated. For comparison between the two tests, the AUC values were compared using the z-test. Statistical analyses were performed in XLSTATPM (version 2017; XLSTAT; Addinsoft, New York, USA).

### Ethics, funding, and potential conflicts of interest.

Approval of the institutional review board was obtained (EK 1156/2016). The study was done in accordance with the Declaration of Helsinki. This research did not receive any funding. The authors have no competing interests to declare.

## Results

### Patient demographic data and infection characteristics

A total of 101 patients (63 women), fulfilled the inclusion criteria. The median age was 71 years (22–91). Previously performed surgeries were 53 total hip arthroplasties, and 48 total knee arthroplasties.

Finally, 29 patients were diagnosed with PJI and 72 cases were classified as aseptic failure according to the MSIS criteria. In 1 of the 29 septic cases, a PJI was diagnosed due to a sinus tract, 2 positive cultures with a phenotypically identical Staphylococcus aureus, and positive minor criteria (permanent section: Type 2, elevated CRP). In 10 of the septic cases, at least 3 minor criteria were fulfilled but no major criterion. In the remaining 18 septic cases, 2 or more periprosthetic positive cultures with the phenotypically identical organisms plus positive minor criteria were present. None of the criteria was positive in 53 of the 72 aseptic cases. In 17 patients, only 1 criterion of the MSIS criteria was fulfilled. In the remaining 2 cases, 2 minor criteria were positive.

### Performance of diagnostic tests

**Figure 1.**
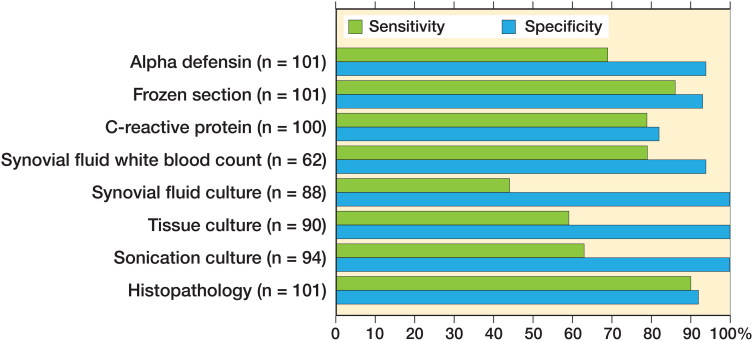
Sensitivity (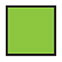) and specificity (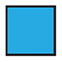) of different tests in diagnosing PJI when using the MSIS criteria.

The median CRP (n = 100) was 5.7 mg/L (0.03–252). The sensitivity and specificity of serum CRP was 79% (CI 61–90) and 82% (CI 71–89), respectively (Figure 1). The median WBC count (n = 62) in the synovial fluid was 5,167 cells per µL (< 1.0–55,140). The sensitivity and specificity of synovial fluid WBC count was 79% (CI 59–91) and 94% (C: 79–99), respectively. The sensitivity of synovial fluid culture (n = 88), tissue culture (n = 90), and sonication (n = 94) was 44% (CI 28–63), 59% (CI 41–74), and 63% (CI 44–78), respectively. The specificity was, in all 3 different culture methods, 100% (CI 93–100).

The most commonly isolated microorganism was *Staphylococcus aureus* (n = 5), followed by coagulase negative Staphylococci (n = 4) and *Escherichia coli* (n = 3) (Table 1). 9 septic cases were culture negative.

**Table 1.** Distribution of microorganisms

Isolated pathogens	Conventional culture (n = 29)
*Staphylococcus aureus*	5
Coag. negative Staphylococci	4
*Escherichia coli*	3
*Cutibacterium acnes*	1
*Streptococcus agalactiae*	1
*Enterococcus faecalis*	1
*Finegoldia magna*	1
*Actinomyces neuii*	1
*Parvimonas micra*	1
*Moraxella osloensis*	1
*Candida albicans*	1
Negative culture	9

Overall, the sensitivity, specificity, accuracy, positive predictive value, negative predictive value, positive likelihood ratio, and negative likelihood ratio of the permanent section were 90% (CI 73–97), 92% (CI 83–96), 91% (CI 86–97), 81% (CI 68–95), 96% (CI 91–100), 11 (CI 5–23), and 0.11 (0.04–0.33), respectively.

### Performance of frozen sections and alpha defensin

Of the 284 samples from the 101 cases, 68 were positive for infection (n = 68 of 284 [24%]). In 30 cases (n = 30 of 101), at least 1 frozen section sample was positive. Overall percentage agreement between frozen section and definitive histology was 98.9% (CI 97.8–100%). A Cohen’s kappa of 0.97 (CI 0.94–1.0) indicated a near perfect agreement between frozen and permanent section. The positive and negative percentage agreement was 96% (CI 88–99) and 100% (CI 98–100), respectively. 3 samples showed a discrepancy between the results of frozen and permanent sections. All 3 tissue samples showed a negative frozen section, while the permanent section revealed an infection (all Type 3 according to Krenn classification). There was no statistically significant difference between the AUCs of the frozen section and the permanent section (p = 0.7).

The frozen sections showed 25 true positive, 4 false positive, 67 true negative, and 5 false negative test results (Table 2).

**Table 2.** Performance of the frozen section and alpha defensin using MSIS criteria for diagnosis of PJI

Performance	Frozen section	Alpha defensin
Sensitivity (%)	86.2 (68.7–95.0)	69.0 (50.6–82.8)
Specificity (%)	93.1 (84.3–97.3)	94.4 (86.0–98.2)
Accuracy (%)	91.1 (85.5–96.6)	87.1 (80.6–93.7)
Positive predictive value (%)	83.3 (70.0–96.7)	83.3 (68.4–98.2)
Negative predictive value (%)	94.4 (89.0–99.7)	88.3 (81.1–95.5)
Positive likelihood ratio	12.4 (5.3–29.3)	12.4 (4.6–33.2)
Negative likelihood ratio	0.15 (0.06–0.37)	0.33 (0.19–0.57)
Area under the curve	0.90 (0.83–0.97)	0.82 (0.73–0.91)

The 95% confidence interval is presented in parentheses

### False negative and false positive results

10 cases with false negative results (frozen section [n = 4], alpha defensin [n = 9]) were found. In 3 patients (Table 3: Patients 2, 3, and 10), both diagnostic methods were negative while a PJI with at least 2 phenotypically identical microorganisms was present. In 6 patients with PJI (Patients 1, 4, 5, 7, 8, and 9), the alpha defensin test was negative, while the frozen section showed an infection. Moreover, in the frozen or permanent section of another septic case (Patient 6), no infection was found, whereas alpha defensin was positive and *Cutibacterium acnes* was identified in culture analysis (2/4 positive tissue cultures, positive sonication) (Table 3).

**Table 3.** False negative and false positive alpha defensin test or frozen section based on Musculoskeletal Infection Society (MSIS) criteria

Patient	Sex	Age	Joint	MSIS	Alpha defensin	Frozen section	Permanent section^a^	Sinus tract	CRP (mg/L)	WBC (cells/µL)	PMN (%)	Culture^b^	Antibiotics
False negatives													
1	M	59	Knee	PJI	Negative	Positive	Type 3	No	11.2	1,456	67	*Staph. epidermidis*	No
2	F	78	Hip	PJI	Negative	Negative	Type 3	No	6.6	645	54	*Enterococcus faecalis*	No
3	M	81	Hip	PJI	Negative	Negative	Type 1	No	23.0	ND	ND	MRSA	No
4	M	44	Hip	PJI	Negative	Positive	Type 2	No	65.1	91,343	92	No growth	No
5	M	22	Hip	PJI	Negative	Positive	Type 3	No	45.0	44,853	91	No growth	No
6	F	86	Hip	PJI	Positive	Negative	Type 1	No	17.5	12,987	86	*Cutibacterium acnes*	No
7	F	82	Hip	PJI	Negative	Positive	Type 2	No	40.2	26,640	97	*Staph. sacchrolyticus*	No
8	F	71	Knee	PJI	Negative	Positive	Type 3	No	46.6	14,638	82	No growth	No
9	F	83	Knee	PJI	Negative	Positive	Type 2	No	160.0	5,068	87	No growth	Yes
10	F	61	Hip	PJI	Negative	Negative	Type 1	No	8.9	ND	ND	*Moraxella osloensis*	No
False positives													
11	F	71	Hip	AF	Positive	Positive	Type 3	No	4.0	ND	ND	No growth	No
12	F	60	Hip	AF	Positive	Positive	Type 2	No	2.9	< 1.0	–	No growth	No
13	F	69	Knee	AF	Positive	Negative	Type 1	No	5.2	2,212	88	No growth	No
14	F	71	Hip	AF	Negative	Positive	Type 2	No	2.6	16,420	71	No growth	No
15	M	74	Hip	AF	Negative	Positive	Type 2	No	1.6	< 1.0	–	No growth	No
16	F	69	Hip	AF	Positive	Negative	Type 1	No	11.8	< 1.0	–	No growth	No
17	M	45	Knee	AF	Negative	Positive	Type 3	No	3.4	< 1.0	–	No growth	Yes

a According to Krenn and Morawietz classification (Morawietz et al.^2009^).

b 2 or more periprosthetic cultures grew phenotypically identical organisms. None of these patients showed bacterial growth in only 1 sample (synovial fluid, tissue, sonication fluid).

PJI = periprosthetic joint infection; AF = aseptic failure; CRP = serum C-reactive protein; WBC = synovial fluid leukocyte count, PMN = polymorphonuclear neutrophils.

On the other hand, using the MSIS criteria, 7 cases were classified as false positive (frozen section [n = 5], alpha defensin [n = 4]). In 2 cases (Patients 15 and 17), only the frozen and permanent sections were positive, while no other MSIS criterion was fulfilled. In another patient (Patient 16) only the alpha defensin test was positive. These 3 cases were categorized as true false positive cases. In 2 patients (Patients 11 and 12), the positive results of frozen section and the alpha defensin test were regarded as potentially true positive. Both cases revealed a positive histopathology (Krenn classification: Type 2 [Patient 11], Type 3 [Patient 12]). In another patient with positive frozen section (Patient 14), only 2 minor criteria were fulfilled. Therefore, no PJI was present according to the MSIS criteria. Nevertheless, the synovial fluid WBC count was elevated (16,420 cells/µL) and the histopathology showed an infection as well (Type 2). The 3 latter cases could therefore be undetected infections when using the MSIS criteria (Ochsner et al. 2016, Renz et al. 2018).

Moreover, the positive result of 1 alpha defensin test (Patient 13) is unclear. In this patient, a borderline leukocyte count (2,212 cells/µL) and elevated percentage of polymorphonuclear neutrophils (88%) was observed, while all other criteria were negative. However, both results (alpha defensin and leukocyte count) were categorized as false positive.

### Comparison between frozen sections and alpha defensin

**Figure 2.**
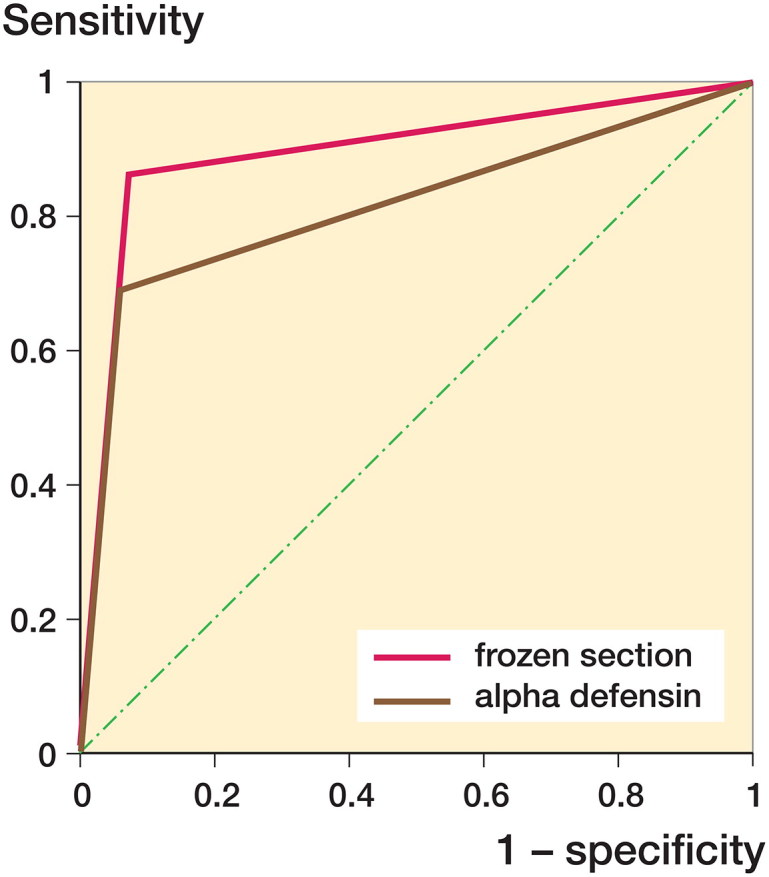
Receiver operating characteristic curves for diagnostic accuracy of periprosthetic joint infection based on the alpha defensin lateral flow test and the frozen section when using the MSIS criteria. There is a statistically significant difference between the two ROC curves (p = 0.006).

The AUCs of frozen section and the alpha defensin test were 0.90 (CI 0.83–0.97) and 0.82 (CI 0.73–0.91), respectively (Figure 2). The difference of both AUCs was 0.023. Of note, the AUCs of the frozen section and the alpha defensin test showed a statistically significant difference (p = 0.006).

### Intraoperative performance

Preoperatively, only 8 of the 29 PJI cases were classified as septic (Table 4). Among these septic cases, 21 did not meet the criteria for PJI preoperatively. 4 did not fulfill any criterion before revision surgery, 4 were positive for only 1 minor criterion, and 13 cases had 2 positive minor criteria. Among the 21 septic cases, the intraoperative alpha defensin test was positive in 13 and frozen section was positive in 17 cases. There was no statistically significant difference between the two tests in intraoperative diagnosis (Fisher’s exact test, p = 0.3).

**Table 4.** Preoperative and postoperative diagnosis of PJI according to the MSIS criteria and intraoperative diagnosis by using alpha defensin lateral flow test and frozen section. Values are frequency (percentage)

	Patients (n)	Preop.PJI	Postop.PJI	Intraoperative positive	
				alpha defensin test	frozen section
Minor criteria					
0 positive	61	0 (0)	4 (7)	3 (75)	3 (75)
1 positive	19	0 (0)	4 (21)	1 (25)	2 (50)
2 positive	13	0 (0)	13 (100)	9 (69)	12 (92)
3 positive	7	7 (100)	7 (100)	6 (86)	7 (100)
Major criterion	1	1 (100)	1 (100)	1 (100)	1 (100)

Minor criterion (serum CRP, synovial fluid leukocyte count, or percentage of polymorphonuclear neutrophils, synovial fluid culture). Major criterion (sinus tract). PJI = periprosthetic joint infection.

Of the 57 cases with postoperative aseptic failures not fulfilling a single positive preoperative criterion, 4 had a positive frozen section (Table 3: Patients 11, 12, 15, 17) and 2 had a positive alpha defensin test (Patients 11 and 12). 15 cases diagnosed with aseptic failure met 1 preoperative positive criterion for PJI, of whom 1 patient had a positive frozen section (Patient 14) and 2 were positive for alpha defensin test (Patients 13 and 16).

## Discussion

The preoperative test results showed no definitive evidence of infection in three-quarters of the cases that actually qualified for infection based on postoperative MSIS criteria. When such a diagnosis (PJI, aseptic failure) cannot be made preoperatively, a rapid and accurate screening test is required to exclude PJI intraoperatively. According to the clinical practice guidelines supported by the American Academy of Orthopedic Surgeons, frozen sections can be useful in ruling in PJI intraoperatively (Della Valle et al. 2011, Parvizi et al. 2013). In addition, attention has also been paid to the alpha defensin lateral flow test to distinguish between infection and aseptic failure after a total joint replacement. Therefore, we evaluated the performance of the alpha defensin test and frozen section for ruling in PJI intraoperatively. To date, no comparative study of the performance of these diagnostic tests has been reported.

Our results show that among preoperative cases with ambiguous diagnosis of infection (n = 21), the intraoperative frozen section and the alpha defensin test were able to confirm PJI in 17/21 and 13/21 of the cases respectively, suggesting that both these methods are reliable in the diagnosis of infection intraoperatively. However, frozen sections yielded very good diagnostic accuracy with a high sensitivity and specificity in diagnosing PJI (when analyzed by an experienced pathologist). There was no statistically significant difference in the ROC curves between frozen and permanent sections. Additionally, a near perfect agreement (Cohen’s kappa = 0.97) between the two histopathological analyses was shown. Overall, a 99% concordance of all cases could be illustrated, which is in line with the reported rates ranging from 95% to 98% in the literature (Wong et al. 2005, Stroh et al. 2012, Kwiecien et al. 2017). The very low discrepancy (1%) usually occurs due to differences in the quality of the sections and samplings. In the hands of experienced pathologists, frozen sections are as reliable as definitive histology and the technique scores in terms of cost-effectiveness, simplicity, and timely results.

In addition, the frozen sections outperformed the alpha defensin test in our study. The alpha defensin test showed a lower sensitivity (69%) when compared with frozen section (86%), and a statistically significant difference in the ROC curves between the two tests. As described by Renz et al. (2018), the alpha defensin test therefore does not appear as an appropriate intraoperative PJI screening test. However, in situations in which the frozen section is unavailable (for example unavailable experienced pathologist) and/or a PJI cannot be confirmed or excluded preoperatively, the alpha defensin test may be a useful adjunct, especially as a confirmatory test, due to its high specificity.

Limitations of this study include, first, that the interpretation of the frozen and permanent sections is pathologist dependent and may be influenced by the results of other diagnostic tests. To limit this potential bias, analysis and interpretation is blinded and independent at our institution. Second, the tissue sampling is surgeon dependent and can result in selection bias by choosing samples with obvious presence/absence of infection, thus possibly altering the performance of frozen sections. Third, the number of collected tissue samples for frozen section varied between 1 and 8 per patient, which might have affected the final performance (higher numbers increase the sensitivity at the cost of specificity (Athanasou et al. 1995). In addition, in few cases, not all required test results were available for the infection evaluation when using the MSIS criteria (Figure 1), which is the reality in clinical routine (Bonanzinga et al. 2017). Finally, although MSIS criteria are considered the gold standard in diagnosing PJI, these criteria may miss some patients with PJI, especially infections caused by low-virulence organisms (Kwiecien et al. 2017, Renz et al. 2018).

In conclusion, the frozen section technique showed high performance and a near perfect concordance with the definitive histology, thus strengthening its position as an appropriate intraoperative PJI screening test in diagnosing PJI, especially when the results of preoperative tests are not interpretable. Nevertheless, some institutions do not have the opportunity to analyze tissue samples intraoperatively. In such cases, the alpha defensin test shows great advantage in confirming the diagnosis of PJI. Although sensitivity was lower compared with frozen sections, this test is highly specific and delivers timely results, thus allowing quick decision-making during surgery.
